# Interpretation of Autosomal Recessive Kidney Diseases With “Presumed Homozygous” Pathogenic Variants Should Consider Technical Pitfalls

**DOI:** 10.3389/fped.2020.00165

**Published:** 2020-04-17

**Authors:** Haiyue Deng, Yanqin Zhang, Yong Yao, Huijie Xiao, Baige Su, Ke Xu, Na Guan, Jie Ding, Fang Wang

**Affiliations:** Department of Pediatrics, Peking University First Hospital, Beijing, China

**Keywords:** autosomal recessive, genetic counseling, homozygous variant, kidney, uniparental disomy

## Abstract

**Background:** A false interpretation of homozygosity for pathogenic variants causing autosomal recessive disorders can lead to improper genetic counseling. The aim of this study was to demonstrate the underlying etiologies of presumed homozygous disease-causing variants harbored in six unrelated children with five different genetic renal diseases when the same variant was identified in a heterozygous state in only one of the two parents from each family using direct sequencing.

**Methods:** Peripheral blood genomic DNA samples were extracted. Six short tandem repeats were used to verify the biological relationships between the probands and their parents. Quantitative PCR was performed to detect mutant exons with deletions. Single nucleotide polymorphism analysis and genotyping with polymorphic microsatellite markers were performed to identify uniparental disomy (UPD).

**Results:** Each proband and his/her parents had biological relationships. Patients 2, 4, and 6 were characterized by large deletions encompassing a missense/small deletion in *DGKE, NPHP1*, and *NPHS1*, respectively. Patients 1 and 5 were caused by segmental UPD in *NPHS2* and *SMARCAL1*, respectively. In patient 6, maternal UPD, mosaicism in paternal sperm or *de novo* variant in *NPHP1* could not be ruled out.

**Conclusions:** When a variant analysis report shows that a patient of non-consanguineous parents has a pathogenic presumed homozygous variant, we should remember the need to assess real homozygosity for the variant, and a segregation analysis of the variants within the parental DNAs and comprehensive molecular tests to evaluate the potential molecular etiologies, such as a point variant and an overlapping exon deletion, UPD, germline mosaicism and *de novo* variant, are crucial.

## Introduction

The routine clinical use of advanced genomic technologies has proven useful in the diagnosis of hereditary kidney diseases. For example, ~30% of children who received kidney transplantation had a genetic kidney disease (1). Therefore, accurate genetic counseling is becoming increasingly important for the effective implementation of personalized medicine, especially for the assessment of recurrence risk. Identifying the mode of inheritance is necessary for genetic counseling and the analysis of recurrence risk. It had been reported that autosomal recessive inheritance, which is caused by homozygous or compound heterozygous pathogenic variants in the alleles of homologous chromosomes, was most common among monogenic kidney diseases (2). True causative-gene homozygous variants are more likely to occur in consanguineous families (3–5). By contrast, presumed homozygous disease-causing variants that are detected by standard DNA sequencing can be a result of a point variant and an overlapping exon deletion, uniparental isodisomy (UPD), or allele dropout owing to single-nucleotide polymorphisms at primer sequences (6), which may not always be provided in variant analysis reports and in turn leads to the misdiagnosis of homozygous variants and then improper genetic counseling. Different causes of presumed homozygosity have different carrier status, leading to different risks of disease recurrence in a proband's siblings. Therefore, medical practitioners and genetic counselors should be aware of the need to assess real homozygosity for variants causing autosomal recessive conditions.

Some patients with autosomal recessive kidney diseases due to UPD have been reported (7–17). However, to our knowledge, patients with autosomal recessive kidney disorders and presumed homozygous disease-causing variants caused by compound heterozygosity of a point variant and an overlapping exon deletion have not been reported elsewhere. In the present study, we report six unrelated probands (from non-consanguineous families) with five different genetic renal diseases and presumed homozygous disease-causing variants resulting from a large deletion encompassing a missense/small deletion, segmental UPD, and mosaicism in paternal sperm/*de novo* variant in the causative-gene. Our results highlight the benefit of comprehensive molecular tests to distinguish real homozygosity from presumed homozygosity, which helps medical practitioners and genetic counselors to provide effective personalized management of autosomal recessive diseases.

## Materials and Methods

### Patients

**Figure 1:**
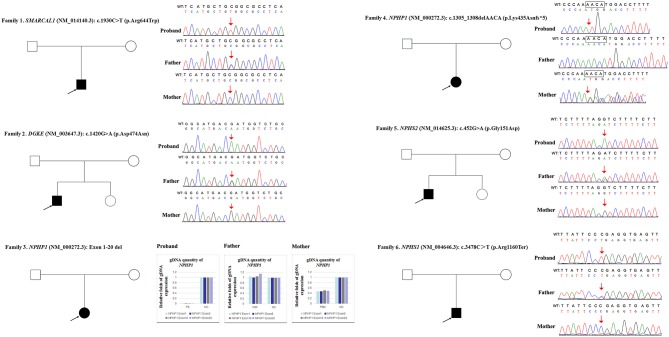
Variations detected in 6 probands and their parents. The filled black squares and circles indicate the individuals with kidney diseases, and the unfilled squares and circles indicate the individuals without renal phenotypes. The black arrows indicate the probands. The red arrows or red arrows and rectangles indicate the variations. WT, normal sequence; NC, normal control.

Over the past 20 years, our division has developed a cohort of 850 patients with a genetic diagnosis of kidney disease that was detected by direct sequencing or next generation sequencing (NGS). Among these patients, our attention was caught by six unrelated patients (0.7%) who seemed to have homozygous disease-causing variants, but only one non-consanguineous parent of each case was confirmed as a carrier of the same variant by Sanger sequencing (Figure 1). Since patient 1 was clinically diagnosed with Schimke immuno-osseous dysplasia, his entire coding exons of *SMARCAL1* were analyzed by using conventional PCR and Sanger sequencing, and the genetic etiologies of patients 2–6 were analyzed by using targeted NGS panel (including 504 hereditary kidney diseases genes, see Supplementary Material) or whole exome sequencing. The clinical and molecular characteristics of these six children were presented as follows and summarized in Table 1. The criteria that were used for considering variants as disease-causing were the same as those we described previously (21).

**Table 1:** General information of six patients.

**Patient**	**Onset age**	**Gender**	**Diagnosis**	**Gene**	**Nucleotide change**	**Predicted effect on protein**	**Zygosity (Segregation)**	**ACMG**		**Ultimate etiology**
								**Classify sequence variants**	**Interpretation**	
1	4	Male	Schimke immunoosseous dysplasia	*SMARCAL1*	1930C>T	p.Arg644Trp (18)	Homozygosity (F)	PS1, PM1, PM2, PP3, PP4	Pathogenic	Paternal UPD
2	2.4	Male	Thrombotic microangiopathy	*DGKE*	1420G>A	p.Asp474Asn	Homozygosity (F)	PM1, PM2, PM3, PP3	Likely pathogenic	An overlapping exon deletion (entire *DGKE* gene)
3	12.8	Female	CKD stage 4 in kidney hypoplasia	*NPHP1*	Exon 1-20 del ^a^ (19)	–	Homozygosity (M)	–	–	Maternal UPD or mosaicism in paternal sperm or *de novo* variant
4	8	Female	CKD stage 4 in kidney hypoplasia	*NPHP1*	1305_1308delAACA	p.Lys435Asnfs*52	Homozygosity (M)	PVS1, PM2, PM3	Pathogenic	An overlapping exon deletion (entire *NPHP1* gene)
5	6.3	Male	Steroid-resistant nephrotic syndrome	*NPHS2*	452G>A	p.Gly151Asp	Homozygosity (F)	PM1, PM2, PP3, PP4	Likely pathogenic	Paternal UPD
6	Postnatal day	Male	Congenital nephrotic syndrome	*NPHS1*	3478C>T	p.Arg1160Ter (20)	Homozygosity (F)	PVS1, PS1, PM2, PP3	Pathogenic	An overlapping exon deletion (*NPHS1* exon 23-29)

*F, father; M, mather; CKD, chronic kidney diseases; Accession no: DGKE(OMIM#601440), NM_003647.3. NPHP1(OMIM#607100), NM_000272.3. NPHS1(OMIM#602716), NM_004646.3. NPHS2(OMIM#604766), NM_014625.3. SMARCAL1(OMIM#606622), NM_014140.3. a: The variation in patient 3 was not suitable for ACMG recommendations, however, it has been reported to be pathogenic*.

### Patient 1

A 7-years old boy was admitted to our hospital because of 4 months of edema and 2 months of proteinuria. An obvious height deficit had continued from 2.5 years of age. He had a characteristic phenotype: short stature, barrel chest, short neck, scoliosis, and café-au-lait spots. The laboratory investigation showed hypoalbuminaemia (25.1 g/L), hypercholesterolaemia (7.78 mmol/L), significant proteinuria (3.16 g/24 h), T cell immunodeficiency (the percentages of peripheral blood T and B lymphocytes were 19 and 67%, respectively, and the total T lymphocytes were 124/μL) and normal renal function. A skeletal X-ray demonstrated first lumbar vertebral dysplasia (small vertebral body and narrow intervertebral space). The diagnosis of Schimke immuno-osseous dysplasia was made based on the clinical data.

### Patient 2

An 8.3-year-old boy was referred to our hospital due to intermittent proteinuria of ~6 years' duration. At the age of 2.4 years old, he was diagnosed as primary nephrotic syndrome by the findings of edema, hypoalbuminaemia (26.8 g/L), hypercholesterolaemia (12.65 mmol/L), and significant proteinuria (1.5 g/24 h). Prednisolone therapy was unable to induce remission, whereas proteinuria responded to prednisolone and cyclosporine therapies. These drugs were discontinued with continuous negative proteinuria. Beginning at 4 years, whenever proteinuria was relapsed, thrombocytopaenia (29~40 × 10^9^/L), anemia (hemoglobin was 72 g/L), and elevated levels of serum creatinine (83~171 μmol/L) were detected. Gross hematuria and hypertension were also observed once. These abnormalities resolved with combination oral prednisolone/methyl-prednisolone and cyclosporine/tacrolimus therapies, diuretics and antihypertensive treatment. On admission at 8.3 years, a physical examination showed no abnormal signs. Laboratory findings showed hematuria, moderate proteinuria (43.4 mg/kg/d), renal dysfunction (serum creatinine was 105 μmol/L, serum urea was 21.4 mmol/L, and the estimated glomerular filtration rate was 39.8 ml/min/1.73 m^2^), and normal hemoglobin, platelet counts, serum albumin, liver enzymes, bilirubin, and coagulation data. The available complement evaluation revealed normal plasma levels of complement C3, C4, factor H and ADAMTS-13 activity, and the patient was negative for anti-complement factor H antibodies. A renal biopsy revealed thrombotic microangiopathy. Therefore, he was eventually diagnosed with thrombotic microangiopathy with an initial manifestation of nephrotic syndrome. He was started on angiotensin converting enzyme inhibitors and angiotensin receptor blockade therapy. In the third week of hospitalization, he had normal renal function (serum creatinine was 38 μmol/L).

### Patient 3

A 13.5-year-old girl was referred to our clinic for renal dysfunction. At 12.8 years of age, because of fatigue, she was found to have renal dysfunction (serum creatinine was 314–391 μmol/L) without proteinuria that was detected by the dipstick test, anemia (88–94 g/L), and hyperparathyroidism (parathyroid hormone was 226.4 pmol/L). The renal ultrasound showed that the bilateral kidney sizes were 8.0 × 3.7 cm (left) and 8.0 × 3.1 cm (right), as well as an enhanced echogenicity of the renal parenchyma. The renal biopsy indicated mesangial proliferative glomerulonephritis and focal glomerular sclerosis. She did not have an intellectual disability, hearing loss or ocular lesion. At the age of 13.5 years, her serum creatinine was 434 μmol/L. She was diagnosed with a chronic kidney disease (CKD) of stage 4 in kidney hypodysplasia.

### Patient 4

An 11-year old girl was admitted to our hospital for renal dysfunction. Laboratory studies showed abnormal renal function (serum creatinine was 251–280 μmol/L, blood urea nitrogen was 17–17.6 mmol/L, and the glomerular filtration rate was measured as 27 ml/min/1.73 m^2^ by renal dynamic imaging) and anemia (80–94 g/L). Neither hematuria or proteinuria were detected by the urine dipstick test. The renal ultrasound demonstrated that the length of the bilateral kidneys was 8.0 cm and the renal parenchyma had an enhanced echogenicity. Magnetic resonance imaging revealed a cyst in the left kidney with a diameter of 0.2 cm. No ocular or hearing lesions were involved. She was diagnosed with a CKD of stage 4 in kidney hypoplasia.

### Patient 5

A 6-years old boy was referred for proteinuria. He was initially diagnosed with primary nephrotic syndrome according due to edema, hypoalbuminaemia (12.4 g/L), hypercholesterolaemia (10.6 mmol/L), and significant proteinuria (140.5 mg/kg/d). Steroid therapy, pulse therapy of methyl-prednisolone, cyclophosphamide, and mycophenolate mofetil were unable to induce remission. A renal biopsy revealed focal segmental glomerulosclerosis.

### Patient 6

A 2.7-year-old boy was referred for proteinuria. He was born at 36^+3^ weeks of gestation by spontaneous delivery. On admission for neonatal pneumonia, he was found to have proteinuria (3+, detected by the dipstick test), hypoalbuminaemia (10–13.2 g/L) and hypercholesterolaemia (8.2–9.2 mmol/L). A diagnosis of congenital nephrotic syndrome was made. On admission at 2.7 years, he was found to have renal dysfunction (serum creatinine was 86.9 μmol/L, and the estimated glomerular filtration rate was 40 ml/min/1.73 m^2^).

### DNA Preparation

Genomic DNA (gDNA) samples were extracted from peripheral blood that was collected from the patients and their family members using a FlexiGene DNA Kit (QIAGEN, Germany, 51206). Qubit (Life Technologies, USA) was used for the DNA quantification.

**Figure 2:**
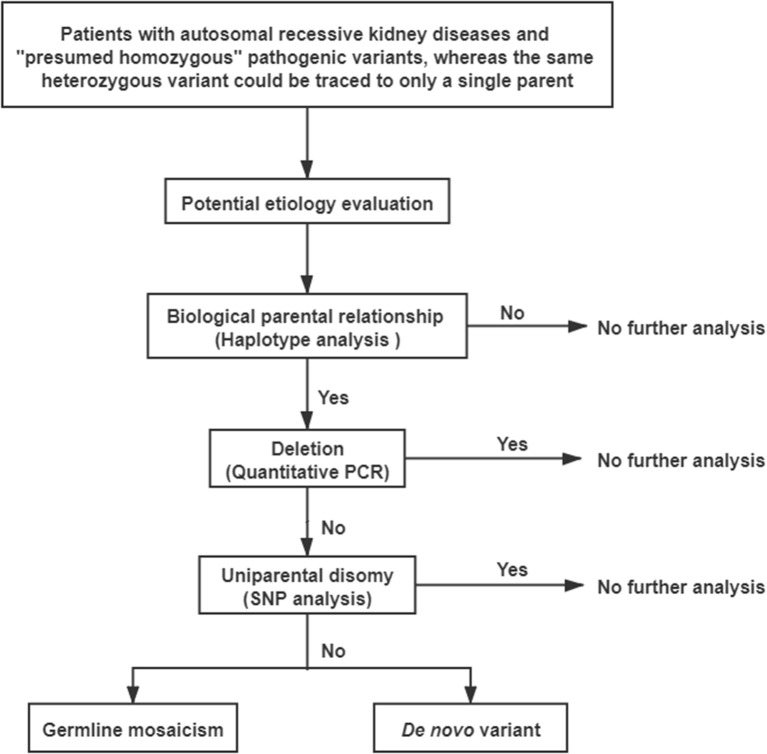
The flowchart to explore potential etiology of pathogenic “presumed homozygous” variants.

A comprehensive genetic evaluation was performed following the flowchart that is shown in Figure 2. All genetic sequences involved in this study were referred to the UCSC Genome Browser and human GRCh37/hg19 (http://genome.ucsc.edu/).

### Haplotype Analysis

Short tandem repeats were used to confirm the biological relationships between probands and their parents. Each forward primer was labeled with FAM fluorescence. Touch-down PCR was used with the following thermal cycling conditions: 94°C for 5 min, 94°C for 30 s, 64°C for 30 s, and 72°C for 45 s, and the annealing temperature reduced 1°C every 2 cycles (from 64 to 58°C), 26 cycles at the final annealing temperature of 58°C, with a final extension at 72°C for 10 min, and termination at 4°C. The amplification products were detected using a 3730XL automatic sequencer (Applied Biosystems).

### Quantitative PCR (qPCR) of gDNA

The qPCR experiment was performed to detect deletions using a Bio-Rad CFX real time PCR system with SYBR Green I qPCR SuperMix (TransGen Biotech, China, AQ131). *GAPDH* or *ACTB* were used as reference genes. The qPCR thermal profile was as follows: 50°C for 2 min, 94°C for 10 min, 94°C for 5 s, and 60°C for 40 s, all for 40 cycles.

### Single Nucleotide Polymorphism (SNP) Analysis

The primers were designed to include the variant site and as many SNP loci as possible. The SNP loci were included when the minor allele frequency was >1% according to the Ensembl website (http://www.ensembl.org). A specific primer pair (5′-CGCCGGCTAATTTTTGTATG and 5′-ACCACTATCTTGCGCTGCTT) was used to analyse the SNP loci that flanked c.1930C>T in *SMARCAL1* in patient 1. The PCR amplification system and program used were the same as described above.

SNP array and genotyping with polymorphic microsatellite markers was available for two patients (3 and 5) and performed using an Infinium Global Screening Array (Illumina, USA).

The targeted NGS, whole exome sequencing and SNP array taken in this study cohort were performed in clinical diagnostic lab which was accredited by authority department in China. However, Sanger sequencing, haplotype analysis and quantitative PCR experiments were performed in our research lab.

## Results

**Figure 3:**
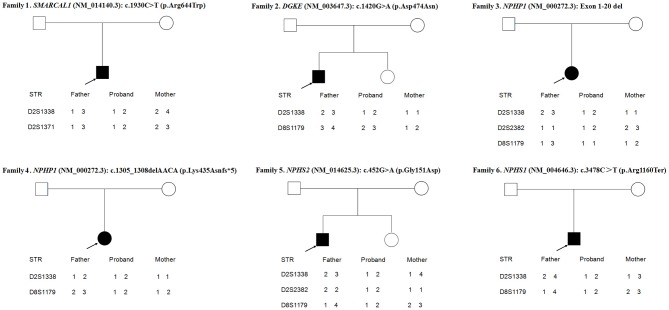
Haplotype analysis of 6 families.

As shown in Figure 3, the loci alleles in different chromosomes demonstrated typical Mendelian inheritance, with paternal and maternal alleles detected in all six patients, confirming the biological relationships between the probands and their parents.

**Figure 4:**
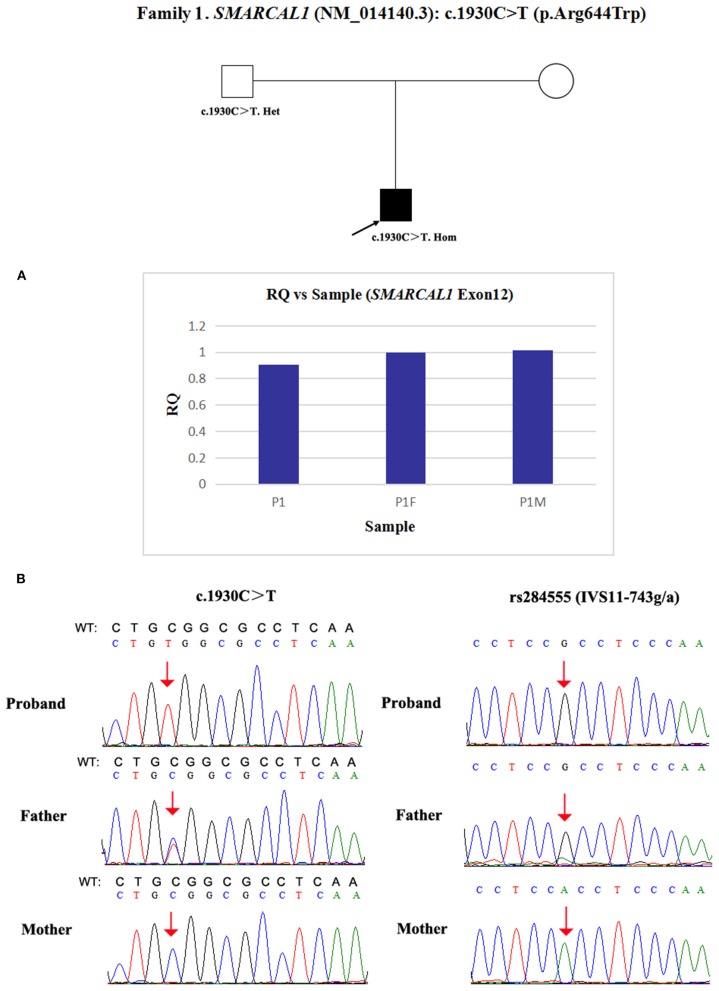
Exploring the potential etiology of presumed homozygous variant in *SMARCAL1* in patient 1. **(A)** qPCR analysis of the exon overlapping the known variant in patient 1 and his parents. RQ, Relative quantity. **(B)** SNP analysis of patient 1 and his parents. The red arrows indicate the SNP and variant. WT, normal sequence.

Due to a confirmed heterozygous missense variant in *SMARCAL1* exon 12 of patient 1's father (Figure 1), he cannot have a deletion in this region. Additionally, the quantities of *SMARCAL1* exon 12 gDNA in patient 1 and his mother were the same as that of patient 1's father (Figure 4A), so a deletion of exon 12 was excluded. Thus, samples from this patient and his parents underwent SNP analysis by PCR amplification and Sanger sequencing. The length of the sequence flanking c.1930C>T (paternal) in *SMARCAL1* was 989 bp, including 8 microsatellite markers. Except for rs284555, other SNPs were not useful for genotyping. rs284555 (IVS11-743g/a) was found to be homozygous IVS11-743g/g in patient 1, while IVS11-743g/g was found in his father and IVS11-743a/a was found in his mother (Figure 4B). The region between IVS11-743g/g to c.1930C>T may be explained as partial paternal UPD of *SMARCAL1*.

**Figure 5:**
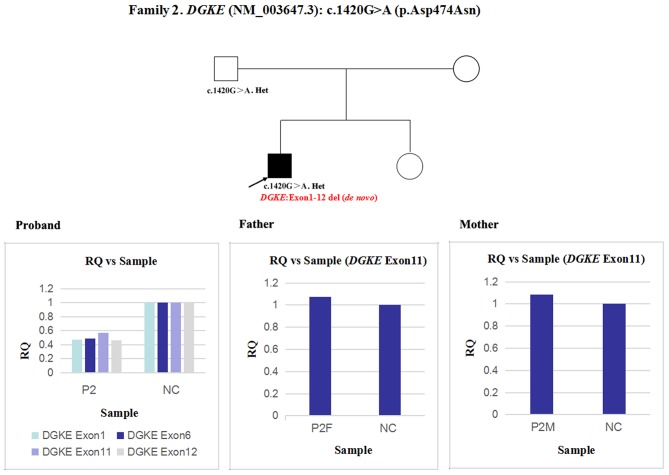
qPCR analysis of the exon overlapping the known variant in patient 2 and his parents; this method was also used to determine the deletion breakpoints. NC, Normal control; RQ, Relative quantity.

Next, qPCR was used to analyse the exons that overlap the known variants to determine whether large deletions existed on another chromosome. It was also used to determine the approximate deletion breakpoints in these genes. As shown in Figure 5, the quantity of *DGKE* exon 11 gDNA in patient 2 was half that of the normal control, while it was normal in his parents. A *de novo* heterozygous deletion may also exist. The breakpoints in *DGKE* were exon 1 and exon 12. In other words, patient 2 had a heterozygous deletion involving the entire *DGKE* gene.

**Figure 6:**
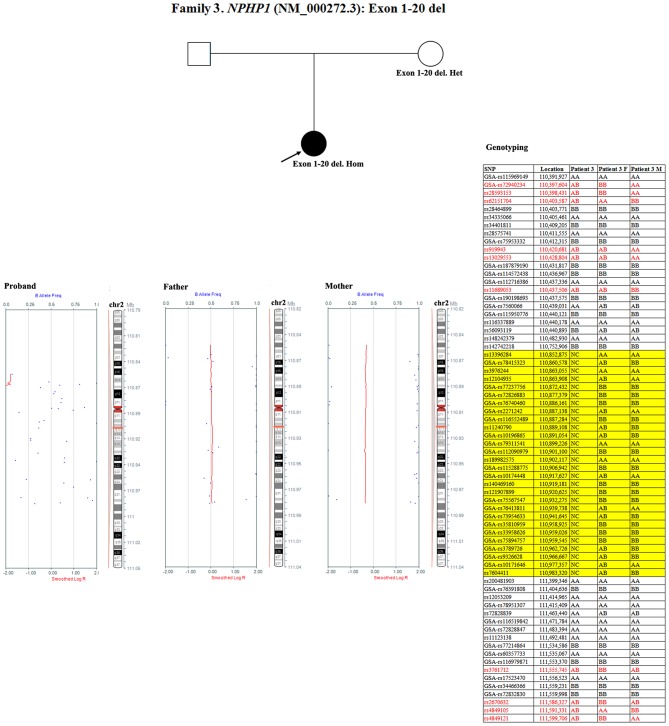
SNP array and genotyping with polymorphic microsatellite markers in patient 3 and her parents. Blue dots with a value of 0, 0.5, or 1 represented SNPs with AA, AB, or BB genotypes, respectively. The red line was used to determine the copy number variation in the targeted region, and it was located at 0.25–0.5 when the targeted region was a single copy. In patient 3, a homozygous deletion near *NPHP1* caused scattered SNP loci distribution. In her mother, the targeted region was a single copy. Partial detailed information on the SNPs near *NPHP1* are also shown. NC referred to no signal displayed. The region in the yellow background represented the deletion (including *NPHP1*). The genotyping in the red font indicated the allele that was inherited from her father.

Patient 3 had a homozygous deletion of the *NPHP1*, whereas only her mother had the same heterozygous deletion (Figure 1). Thus, a SNP array analysis was performed in this family to check for UPD. As shown in Figure 6, because of the limited probes in chromosome 2, the approximate range of the deletion encompassed chr2:110852875 through chr2:110983320. SNP loci near *NPHP1* could not be used to definitively conclude that the two alleles were inherited only from her mother.

**Figure 7:**
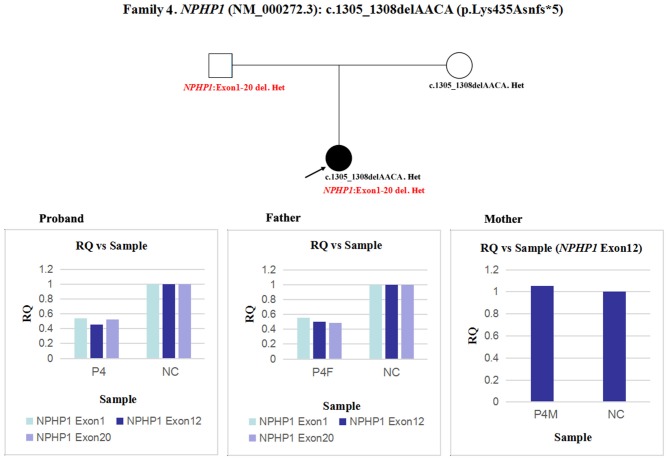
qPCR analysis of the exon overlapping the known variant in patient 4 and her parents, and it was also used to determine the deletion breakpoints. NC, Normal control; RQ, Relative quantity.

In patient 4 and her father, the quantity of *NPHP1* exon 12 gDNA was half that of the normal control (Figure 7). Namely, a heterozygous deletion of *NPHP1* involving exon 12 was in patient 4 and was inherited from her father. The deletion breakpoints in *NPHP1* were exon 1 and exon 20. In other words, patient 4 had a heterozygous deletion involving the entire *NPHP1* gene.

**Figure 8:**
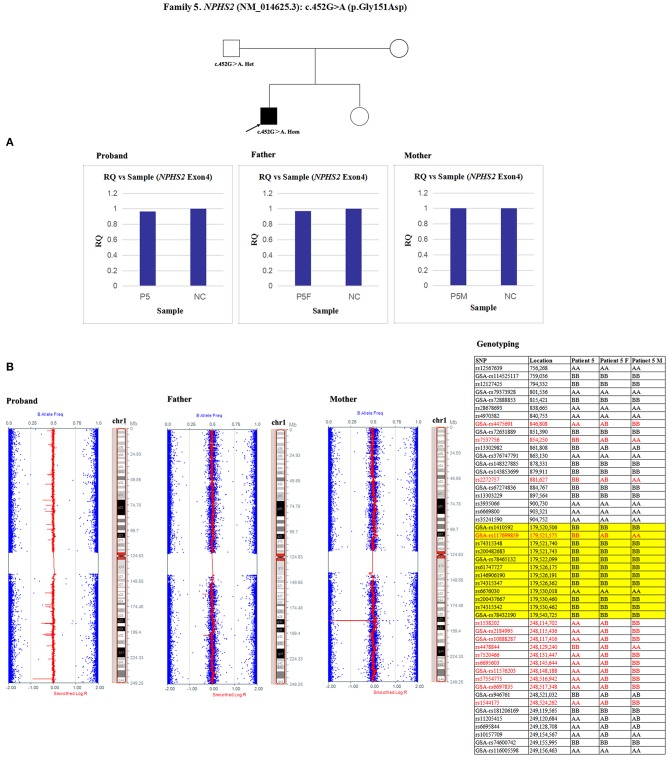
Exploring the potential etiology of a presumed homozygous variant in *NPHS2* in patient 5. **(A)** qPCR analysis of the exon overlapping the known variant in patient 5 and his parents. NC, Normal control; RQ, Relative quantity. **(B)** Detection of uniparental disomy by SNP array and genotyping with polymorphic microsatellite markers in patient 5 and his parents. Blue dots with a value of 0, 0.5, or 1 represented SNPs with AA, AB, or BB genotypes, respectively. The red line was used to determine the copy number variation in the targeted region, and it was normally located near 0.5. In patient 5, two identical copies of almost all SNPs on chromosome 1 were observed, which was in accordance with complete uniparental disomy in chromosome 1. Partial detailed information on the SNPs on chromosome 1p, 1q and *NPHS2* (yellow background) were also shown. The genotyping in the red font could effectively distinguish the origin.

Since qPCR showed that the quantity of *NPHS2* exon 4 gDNA was normal in patient 5 and his parents compared with the normal control (Figure 8A), the possibility of a deletion involving exon four was excluded in this family. However, paternal UPD in chromosome one of patient five was confirmed according to the comparison of parental genotyping with polymorphic microsatellite markers (Figure 8B).

**Figure 9:**
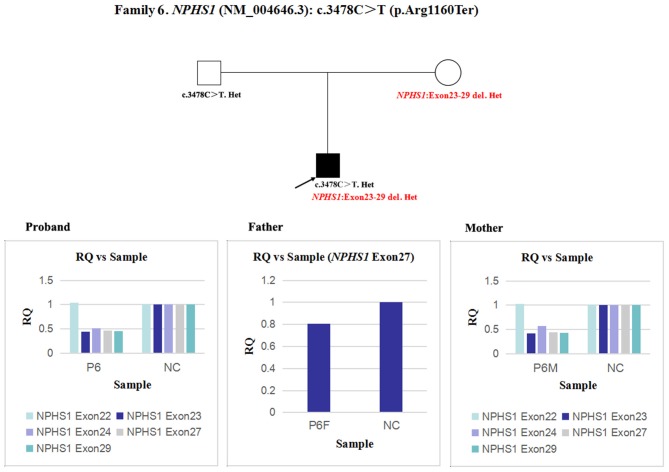
qPCR analysis of the exon overlapping the known variant in patient 6 and his parents, and it was also used to determine the deletion breakpoints. NC, Normal control; RQ, Relative quantity.

In patient 6 and his mother, the quantity of *NPHS1* exon 27 gDNA was half that of the normal control (Figure 9). A heterozygous deletion of *NPHS1* involving exon 27 was observed in patient 6 and was inherited from his mother. The deletion breakpoints in *NPHS1* were exon 23 and exon 29. In other words, patient 6 had a heterozygous deletion involving *NPHS1* exon 23 to exon 29.

## Discussion

In the present study, we identified the etiologies of presumed homozygous disease-causing variants that were harbored in six children with five autosomal recessive kidney diseases. To our knowledge, no compound heterozygosity of a missense/small deletion and an overlapping exon deletion or UPD in *NPHS2* and *SMARCAL1* leading to false homozygosity have been previously reported in renal diseases. Additionally, our observations emphasized two key points in dealing with inherited kidney diseases: (1) A segregation analysis of the variants within parental DNAs is crucial. As discovered here, if the parental samples had not been analyzed, patients 2, 4, and 6 would be misdiagnosed as false homozygosity for causative-gene variants. (2) The genetic findings provided by NGS must be interpreted with caution; if necessary, the NGS data should be re-examined by specialists in the field. Heterozygous large deletions removed more than 9,000 base pairs of the causative gene, and this was detected in patients 2, 4, and 6 using qPCR, but it should be identified by NGS analyzed by specialists.

UPD is defined as an abnormal condition when two homologous chromosomes or chromosomal regions are inherited from a single parent (22). The concept was initially described in 1980 (23), and the first case was reported in 1987 using molecular methods (24). The incidence of UPD on any chromosome was reported to be 1 in 3,500 live births (25). Angelman and Prader-Willi syndromes can arise by inheritance of paternal and maternal UPD in chromosome 15, and this is known by most clinicians (26). The etiology may involve monosomy or trisomy rescue and gamete complementation. Uniparental heterodisomy and uniparental isodisomy are two forms of UPD. The difference relates to whether the alleles that were inherited from one parent were two different or two identical copies (27). Although not necessarily pathogenic, uniparental isodisomy can result in autosomal recessive disorders if the parent is a carrier of a deleterious gene variant (28–30). In recent decades, 3,650 cases of UPD have been identified (http://upd-tl.com/upd.html), whereas there are only 11 reports of autosomal recessive kidney diseases. In the present study, segmental UPD of *SMARCAL1* and *NPHS2* was the disease mechanism of patients 1 and 5, respectively. However, the risk of their siblings being affected is negligible.

There is another potential etiology that may masquerade as homozygous variants. It was described as compound heterozygosity of a point variant and an overlapping exon deletion. Many studies named it “apparent homozygosity” (5, 31). Due to the presence of heterozygous deletion, only one chromosome with the point variant could be amplified, leading to similar effect as with homozygous variant. In a study based on the frequency and molecular etiology behind homozygous variants, 2/75 cases presented with apparent homozygosity (32). In a search for related articles, no relevant studies on kidney diseases were identified, and most reports were associated with cystic fibrosis, which is caused by pathogenic variants in *CFTR* (31, 33). In our study, apparent homozygosity in three different causative genes (*DGKE, NPHP1*, and *NPHS1*) were detected in patients 2, 4, and 6, respectively. A quantitative analysis confirmed a large deletion of one allele in each case. Each parent of patients 4 and 6 carried a pathogenic allele. Therefore, the recurrence risk in the patients' siblings was 25%.

In contrast, the deletion in *DGKE* was not detected in the peripheral blood gDNA of patient 2′s parents. The heterozygous deletion in patient 2 may have been inherited from the maternal germ cell or arisen as a *de novo* variant. Since oocytes are not easily obtained, it is difficult to diagnose germline mosaicism by germ cell sequencing. However, an evaluation of the recurrence risk in the proband's siblings is possible. If his mother has germline mosaicism, the risk in each future pregnancy depends on the proportion of mutant germ cells. Moreover, a *de novo* variant could not be excluded, although it was consistent with the mutant allele that rarely occurred. In this situation, only patient 2′s father carried the pathogenic missense variant, and the patient's siblings had a 50% risk of being a carrier. The recurrence risk could be rare.

In patient 3, SNP loci in the region from chr2:110437575 to chr2:111553370 could not clearly determine the allele's origin. Maternal UPD, mosaicism in paternal sperm, and *de novo* variant in *NPHP1* cannot be excluded. However, compared with the low probability of *de novo* variant that was identical to the existing deletion, the other two possibilities were more likely to occur.

A limitation of this study was that only qPCR was used to quantify the breakpoints in the mutated genes. Array comparative genomic hybridization or copy number variation sequencing can be used to determine the true region of large deletions. Due to the a limited amount of data captured by SNP array in patient 3, the potential etiology of the homozygous deletion remains unclear. Nanopore long-read whole genome sequencing combined with R10 high accuracy mode and high-depth next generation sequencing of sperm DNA can help analyse most SNPs and determine the proportion of mutant germ cells, respectively. However, we had difficulty obtaining peripheral blood and semen samples.

In conclusion, in autosomal recessive kidney disorders, patients were determined to have causative-gene “presumed homozygous” variants, whereas only a single parent was found to harbor the same heterozygous variant using Sanger sequencing. The potential molecular etiologies such as a point variant and an overlapping exon deletion, UPD, germline mosaicism and *de novo* variant should be suspected and analyzed. Furthermore, a very rare condition such as allele dropout caused by SNPs at primers sites also should be considered. Genetic counseling in such conditions necessitates a careful evaluation of genetic results and clinical features of the proband and his/her relatives.

## Data Availability Statement

The data in this study is deposited in GenBank with accession numbers MT009330 to MT009342.

## Ethics Statement

The studies involving human participants were reviewed and approved by The Ethical Committee of Peking University First Hospital (approval number: 20161029). Written informed consent to participate in this study was provided by the participants' legal guardian/next of kin.

## Author Contributions

HD helped design the project, performed the experiments, analyzed the data, composed first draft, and helped make final edits to produce the final manuscript for submission. JD and FW contributed to design the project, revised the manuscript, and helped make final edits to produce the final manuscript for submission. YZ helped design the project, performed the experiments, and analyzed the data. YY, HX, BS, KX, and NG helped provide patients' clinical data. All authors reviewed the manuscript.

## Conflict of Interest

The authors declare that the research was conducted in the absence of any commercial or financial relationships that could be construed as a potential conflict of interest.
