# Supplementary material for: Interpretation of Autosomal Recessive Kidney Diseases With “Presumed Homozygous” Pathogenic Variants Should Consider Technical Pitfalls
Source: Front Pediatr. 2020 Apr 17;8:165. doi: 10.3389/fped.2020.00165 (PMC7180205; doi:10.3389/fped.2020.00165)
Supplement: Supplementary file 1 [file Data_Sheet_1.doc]

**Supplement**

**Gene list in hereditary kidney diseases panel**

*ABCC6, ABCG2, ACAT1, ACE, ACTN4, ADAMTS13, ADCK4, AGL, AGPAT2, AGT, AGTR1, AGXT, AHI1, AIP, AKT1, AKT3, ALDOA, ALDOB, ALG1, ALMS1, ALPL, APC, APOA1, APOE, APOL1, APRT, AQP2, ARHGAP24, ARHGDIA, ARL13B, ARL6, ARX, ASL, ATP6V0A4, ATP6V1B1, ATP7B, ATRX, AURKA, AVPR2, AXIN2, B3GALTL, B9D2, BBS1, BBS10, BBS12, BBS2, BBS4, BBS5, BBS7, BBS9, BCL10, BCS1L, BICC1, BMP4, BMPER, BSCL2, BSND, BUB1B, C3, CA2, CASR, CAV1, CBS, CC2D2A, CCBE1, CCDC28B, CCND1, CD2AP, CD46, CD96, CDC73, CDKN1B, CDKN1C, CEP290, CFB, CFH, CFHR1, CFHR3, CFHR4, CFHR5, CFI, CHD7, CLCN5, CLCN7, CLCNKA, CLCNKB, CLDN16, CLDN19, CNNM2, COA5, COL4A1, COL4A3, COL4A4, COL4A5, COL4A6, COQ2, COQ6, COQ9, COX14, COX6B1, CPT1A, CPT2, CTNS, DGKE, DHCR7, DHODH, DIRC2, DIS3L2, DKC1, DMP1, DYNC2H1, EGF, EIF2AK3, ENO3, ENPP1, EP300, EPO, ERBB3, ERCC6, ERCC8, ESCO2, ETFA, ETFB, ETFDH, EYA1, FAH, FAM123B, FAM20A, FAM20C, FAM58A, FAN1, FANCA, FANCC, FANCD2, FANCE, FASTKD2, FGA, FGF10, FGF23, FGFR2, FGFR3, FH, FLCN, FLNB, FN1, FOXC1, FOXC2, FRAS1, FREM1, FREM2, FXYD2, G6PC, G6PC3, GAA, GALNT3, GALT, GATA3, GBE1, GDNF, GLA, GLI3, GLIS2, GNAS, GNAS-AS1, GPC3, GPC5, GRHPR, GSN, GYG1, GYS1, GYS2, H19, HMGA2, HNF1A, HNF1B, HOGA1, HOXA13, HOXD13, HPRT1, HPS1, HRAS, HSD11B2, HSD17B4, IFNG, IFT122, IFT140, IFT43, IFT80, IKBKAP, IKBKG, INF2, INPP5E, INSL3, INVS, IQCB1, IRF4, ITGA3, ITGA4, ITGB4, JAG1, JAM3, KAL1, KANSL1, KAT6B, KCNJ1, KCNJ10, KCNQ1OT1, KIF1B, KL, KRAS, LAMB2, LAMB3, LCAT, LDHA, LIG4, LMBRD1, LMX1B, LPIN1, LRP4, LYZ, MAFB, MAX, MBTPS2, MEFV, MET, MITF, MKKS, MKS1, MLH3, MLL2, MMAA, MMAB, MMACHC, MMADHC, MNX1, MTHFR, MTR, MTRR, MUC1, MUT, MVK, MYH9, MYO1E, NEK1, NEK8, NF1, NIPBL, NLRP3, NME1, NOTCH2, NPHP1, NPHP3, NPHP4, NPHS1, NPHS2, NR3C2, NRAS, NSD1, NSDHL, OCRL, ODC1, OFD1, OGG1, OSTM1, PAX2, PC, PDGFRL, PDSS2, PEX1, PEX5, PFKM, PGAM2, PGK1, PGM1, PHEX, PHKA1, PHKA2, PHKB, PHKG2, PIGL, PIGN, PIK3CA, PIK3R2, PKD1, PKD2, PKHD1, PLA2G2A, PLCE1, PLEKHM1, PLG, PMM2, POMT1, POR, PORCN, PQBP1, PRCC, PRKAG2, PRKCSH, PROC, PRODH, PROKR2, PRPS1, PTEN, PTPN11, PTPRJ, PTPRO, PTRF, PYGL, PYGM, RAB40AL, RAD51C, RAI1, RECQL4, REN, RET, RNF139, RNU4ATAC, ROBO2, ROR2, RPGRIP1L, RRM2B, RTTN, RXFP2, SALL1, SALL4, SARS2, SCARB2, SCNN1A, SCNN1B, SCNN1G, SDCCAG8, SDHB, SDHD, SEC63, SEMA3E, SERPINH1, SF3B4,SI, SIX1, SIX2, SIX5, SLC12A1, SLC12A3, SLC22A12, SLC22A5, SLC26A3, SLC2A2, SLC2A9, SLC34A1, SLC34A3, SLC36A2, SLC37A4, SLC3A1, SLC4A1, SLC4A4, SLC5A1, SLC5A2, SLC6A19, SLC6A20, SLC7A7, SLC7A9, SLC9A3R1, SMARCAL1, SMARCB1, SMPD1, SOX17, SRCAP, STRA6, STX16, SUCLA2, TCIRG1, TCTN3, TFAP2A, TFE3, TGFBR2, THBD, TLR2, TLR4, TMEM127, TMEM138, TMEM216, TMEM231, TMEM237, TMEM67, TNFRSF11A, TNFSF11, TP53, TP63, TREX1, TRIM32, TRPC6, TRPM6, TSC1, TSC2, TTC19, TTC21B, TTC8, UMOD, UPK3A, UQCRB, UQCRQ, VDR, VEGFA, VHL, VIPAS39, VPS33B, WDPCP, WDR19, WNK1, WNK4, WNT3, WNT4, WNT5A, WT1, XDH, XPNPEP3, XYLT1, XYLT2, ZMPSTE24, ZNF423, CAMK2B, KIF4B, SRCIN1, CAPZA1, ADD2, ARHGAP35, PDLIM1, PTPRE, LLGL1, DYNC1LI1, MYOZ2, PTP4A1, CUBN, CRB2, ITGA8, DSTYK, CD151, WDR73, FGF20, ANLN, ARHGAP4, EMP2, ADD3, KAT2B ,FAT1, NUP160, RHPN1, NEIL1, BMP7, CDC5L, CHRM3, GRIP1, HPSE2, FGF8, FGFR1, PAX8, SEMA3A, TNXB, TBC1D1, TBX18, PAPLN, TUBAL3, TTC25, CHD1L, TRAP1, DCDC2, NXF5, LMNA, KANK1, KANK2, KANK4, MAGI1, RHOXF2B, HP, PLCH1, DHTKD1, NUP85, NUP107, NUP133, NUP93, NUP205, XPO5, ARA55, WFS1, UPB1, CTH, STAR,C1QA, C4A, PTPN22, FCGR2A, FCGR2B, CTLA4, DNASE1, PIGA, PIGT, CISD2, EVC, EVC2, HPD, PNP, SLC17A5, GLB1, NDUFA2*
